# The Roots of *Atractylodes macrocephala* Koidzumi Enhanced Glucose and Lipid Metabolism in C2C12 Myotubes via Mitochondrial Regulation

**DOI:** 10.1155/2015/643654

**Published:** 2015-11-04

**Authors:** Mi Young Song, Seok Yong Kang, Tae Woo Oh, Rethineswaran Vinoth Kumar, Hyo Won Jung, Yong-Ki Park

**Affiliations:** ^1^Department of Rehabilitation Medicine of Korean Medicine, College of Korean Medicine, Dongguk University, Gyeongju 707, Republic of Korea; ^2^Korean Medicine R&D Center, College of Korean Medicine, Dongguk University, Gyeongju 707, Republic of Korea; ^3^Department of Herbology, College of Korean Medicine, Dongguk University, Gyeongju 707, Republic of Korea

## Abstract

The root of *Atractylodes macrocephala* Koidzumi (Atractylodis Rhizoma Alba, ARA) is a Traditional Korean Medicine and has been commonly used for weight control. Mitochondrial dysfunction appears to be a key contributor to insulin resistance, and therefore mitochondrial targeting drugs represent an important potential strategy for the treatment of insulin resistance and obesity. In this study, the authors investigated the regulatory effects of ARA on mitochondrial function with respect to the stimulation of glucose and lipid metabolism in C2C12 myotubes. After differentiating C2C12 myotubes, cells were treated with or without different concentrations (0.2, 0.5, and 1.0 mg/mL) of ARA extract. ARA extract significantly increased the expression of peroxisome proliferator-activated receptor coactivator 1 alpha (PGC1*α*) and the downregulations of its targets, nuclear respiratory factor-1 (NRF-1), transcription factor A (TFAM), and total ATP content in C2C12 myotubes. ARA extract also increased the expressions of PGC1*α* activator and of the metabolic sensors, AMP-activated protein kinase (AMPK), and acetyl-CoA carboxylase and sirtuin (SIRT) 1. Furthermore, it significantly increased glucose uptake by enhancing glucose consumption and subsequently decreased FFA contents and increased carnitine palmitoyltransferase (CPT) 1b expression. Our study indicates that ARA has a potential for stimulating mitochondrial function and energy metabolism in muscle.

## 1. Introduction

Mitochondria play an important role in energy metabolism by activating glucose transport and fatty acid oxidation. Imbalance between energy intake and expenditure leads to mitochondrial dysfunction, which contributes to the pathogeneses of age-associated diseases, such as obesity, insulin resistance, and type 2 (T2) diabetes [1].

Skeletal muscle is a crucial tissue from the perspectives of mitochondrial dysfunction and insulin resistance. Cumulative evidence strongly suggests that changes in mitochondrial function in skeletal muscle are closely related with both insulin resistance and T2 diabetes [2–5]. Furthermore, insulin resistance is highly associated with myocellular lipid accumulation [6, 7] and impaired oxidative capacity of skeletal muscle (caused by mitochondrial dysfunction induced impairment of fatty acid oxidation) and accelerates or directly causes insulin resistance.

Peroxisome proliferator-activated receptor coactivator 1 alpha (PGC1*α*) is a key factor of mitochondrial function. PGC1*α* is considered a master regulator of mitochondrial biogenesis and a potent coactivator of a plethora of transcription factors that impact whole body energy expenditure. Furthermore, PGC1*α* is a coactivator of nuclear transcription factors, such as nuclear respiratory factor-1 (NRF-1) and transcription factor A (TFAM), which are crucially required for mitochondrial gene expression and replication of the mitochondrial genome [8, 9]. In skeletal muscle, two metabolic sensors, AMP-activated protein kinase (AMPK) and sirtuin (SIRT) 1, are known to affect the activity of PGC-1*α* directly via the phosphorylation of AMPK and deacetylation of SIRT1 [8]. The AMPK system is a key player in the regulation of energy balance at both the cellular and whole body levels and is placed centre stage in studies on obesity, diabetes, and metabolic syndrome. In particular, the activation of AMPK in skeletal muscle increases glucose uptake, fatty acid oxidation, and mitochondrial biogenesis by increasing the expressions of genes involved in these pathways [8, 10]. SIRT1, an enzyme that mediates the NAD^+^-dependent deacetylation of target substrates, is a well-known activator of PGC-1*α*, and the two molecules, AMPK and SIRT1, have similar effects on cellular fuel metabolism and mitochondrial function because they regulate each other and share many common target molecules [11].

Recently, the developments of mitochondrial targeting drugs or nutrients for the treatment of insulin resistance, obesity, and type 2 diabetes have opened up new avenues for enhancing health [12]. Several drugs and a handful of natural and nutritional compounds, such as metformin, AMPK activator [13], thiazolidinedione (PPAR*γ* agonist) [14], and resveratrol, SIRT1 activator [15], have been shown to regulate mitochondrial biogenesis and reduce insulin resistance. To date few medicinal plants have been investigated in this context, and, thus, natural products are viewed optimistically as a means of providing agents for the treatment of insulin resistance and its related metabolic diseases. The roots of* Atractylodes macrocephala* Koidzumi (Atractylodis Rhizoma Alba, ARA, Compositae) are used in Traditional Korean Medicine (TKM) for the treatment of gastrointestinal diseases, abdominal pain, and obesity, and it has been shown that ARA extract has anti-inflammatory [16, 17], antiulcer [18], and antitumor effects [19, 20]. Furthermore, the administration of ARA extract to high fat-fed obese rats reduced body weight gain and plasma triglyceride levels [21], and ARA extract has been reported to activate insulin signaling pathways in 3T3-L1 adipocytes [22]. However, the underlying mechanisms responsible for its effects on obesity and insulin resistance have not been studied in depth.

Therefore, in the present study, we investigated whether ARA extract has the ability to regulate glucose and lipid metabolism by regulating mitochondrial function in skeletal muscle cells.

## 2. Materials and Methods

### 2.1. Preparation of ARA Extract

ARA was purchased from Medicinal Materials Company (Kwangmyungdang Medicinal Herbs, Ulsan, Korea) and authenticated by Professor Y.-K. Park, a medical botanist in the Department of Herbology, College of Korean Medicine, Dongguk University, Republic of Korea. ARA extract was prepared using a standard procedure. In brief, dried ARA (200 g) was ground, boiled in purified drinking water for 3 h, filtered through a two-layer Whatman number 3 filter paper, and concentrated under vacuum (yield 26%). The dried powder obtained (ARA extract) was stored at −80°C and dissolved in distilled water prior to assays. The compositional analysis of ARA extracts was performed by a HPLC system (Agilent Technologies 1260 Infinity, USA). Atractylenolide III (Sigma-Aldrich, St. Louis, MO, USA) was used as a standard. Samples were obtained by centrifugation (13,000 g × 3 min) and filtering through a 0.2 *μ*m syringe filter. The samples were determined directly using an HPLC system equipped with a Aminex-87H column (150 mm, 4.6 mm, Bio-Rad, USA) and UV detector. The results are shown in Supplementary Figure 1 in Supplementary Material available online at http://dx.doi.org/10.1155/2015/643654.

### 2.2. Cell Culture and Differentiation

C2C12 cells were purchased from ATCC (CRL-1772: Manassas, VA, USA) and cultured in DMEM (Invitrogen, Grand Island, NY, USA) supplemented with 10% FBS (Invitrogen) and a penicillin/streptomycin mix (Invitrogen). For differentiation, C2C12 myoblasts were plated on 6-well plates and cultured till confluent, and then the medium was changed to differentiation medium (DMEM supplemented with 2% horse serum; Invitrogen) every 24 h for 5 days. Differentiated C2C12 myotubes (C2C12 cells as follows) were then treated with or without different concentrations of ARA or metformin as a reference drug.

### 2.3. MTT Assay

The cytotoxic effect of ARA extract was evaluated using a MTT-based colorimetric viability assay (Roche Diagnostics GmbH, Mannheim, Germany). C2C12 cells (5 × 10^4^ cells/well) were plated in different concentrations of ARA extract in 96-well microtiter plates (Nunc, Roskilde, Denmark) and then cultured for 24 h at 37°C in a 5% CO_2_ incubator. At culture termination, MTT (0.5 mg/mL) solution was added to each well and cultured for 4 h at 37°C in a 5% CO_2_ incubator. Solubilizing solution 100 *μ*L was added to each well, and the plate was allowed to stand overnight in the incubator. The optical densities (OD) of solubilized formazan crystals were measured at 570 nm using a microplate reader (UVM340, Asys Hitech GmbH, Austria).

### 2.4. Western Blotting

Cells were lysed using ice-cold lysis buffer (0.1 mL of 50 mM Tris-HCl (pH 7.2) containing 1% sodium deoxycholate, 0.1% SDS, 0.15 M NaCl, and 1% NP-40), and the so-obtained cell lysates were assayed for protein concentration by Bradford staining. Equal amounts of protein (20 *μ*g/mL) were electrophoresed on 10% SDS-acrylamide gels and transferred to nitrocellulose membranes using an electric transfer system. Nonspecific binding was blocked by treating membranes with 3% skim milk in TBST buffer (5 mM Tris-HCl, pH 7.6, 136 mM NaCl, and 0.1% Tween-20) for 1 h. Blots were incubated for 1 h at room temperature with primary antibody against anti-phospho-AMPK*α* (Thr 172), anti-AMPK*α*, anti-phospho-acetyl-CoA carboxylase (ACC) (Ser79), anti-ACC, anti-SIRT1 (Cell Signaling Technology, Danvers, MA, USA), anti-PGC1*α* (Santa Cruz Biotechnology, Santa Cruz, CA, USA), glucose transporter (GLUT) 4 (Santa Cruz Biotechnology), and anti-*β*-actin (Sigma-Aldrich, St. Louis, MO, USA) and then incubated for 1 h at RT with horseradish peroxidase- (HRP-) labeled anti-mouse IgG (1 : 1000, Santa Cruz Biotechnology), washed with 1x TBST three times, and developed with the ECL Western detection reagents (GE Healthcare Bio-Sciences, Pittsburgh, PA, USA). Protein bands were quantified by densitometry using Image J.

### 2.5. Reverse Transcriptase-Polymerase Chain Reaction (RT-PCR)

Total RNA was isolated from cells using TRIzol reagent, according to the manufacturer's instructions (Gibco-BRL Life Technologies Inc., Grand Island, NY, USA). The cDNA was generated as previously described [23]. PCR reactions were conducted in a mixture containing 2 *μ*L cDNA, 4 *μ*M 5′ and 3′ primers, 10x buffer (10 mM Tris-HCl (pH 8.3), 50 mM KCl, 0.1% Triton X-100, 25 mM MgCl_2_; Takara Bio Ltd., Shiga, Japan), 250 *μ*M of dNTP, and 1 U of Tag polymerase (Takara Bio Inc., Shiga, Japan) under the following conditions: 30 sec at 94°C (denaturation), 30 sec at 60°C (annealing), and 1 min at 72°C (extension) for 30 cycles, followed by a final extension for 5 min at 72°C. The following primers were used: mouse PGC1*α* [sense; 5′-CAC CAA ACC CAC AGA AAA CAG-3′, antisense; 5′-GGG TCA GAG GAA GAG ATA AAG TTG], mouse NRF1 [sense; 5′-AAT GTC CGC AGT GAT GTC C-3′, antisense; 5′-GCC TGA GTT TGT GTT TGC TG-3′], mouse TFAM [sense; 5′-CAC CCA GAT GCA AAA CTT TCA G-3′, antisense; 5′-CTG CTC TTT ATA CTT GCT CAC AG-3′], mouse UCP3 [sense; 5′-CAG ATC CTG CTG CTA AAT-3′, antisense; 5′-GCA TCC ATA GTC CCT CTG TAT-3′], mouse CPT-1b [sense; 5′-CCT CCG AAA AGC ACC AAA AC-3′, antisense; 5′-GCT CCA GGG TTC AGA AAG TAC-3′], and GAPDH primers [sense; 5′-GAC ATC ATA CTT GGC AGG-3′, antisense; 5′-CTC GTG GAG TCT ACT GGT-3′]. GAPDH was used as the internal PCR control. The bands were detected by UV and quantified by densitometry using Image J program.

### 2.6. ATP Content

Total ATP content was determined using ATP calorimetric assay kit (BioVision, Inc., CA, USA). Cells were differentiated for 5 days and then treated with ARA extract or metformin for 24 h. ATP concentration was determined according to the manufacture's protocol; the absorbance was measured at *λ* = 570 nm.

### 2.7. Glucose Consumption Assay

Glucose consumption was assayed using cell culture supernatants. Briefly, C2C12 cells were cultured in 96-well plates and, after reaching confluence, the medium was replaced by phenol red free DMEM adding 11.1 mmol/L glucose and 0.2% BSA. Cells were incubated with ARA extract (0.2, 0.5, or 1 mg/mL), metformin (2.5 mM), or insulin (100 nM) for 24 h. Glucose contents in media were determined using glucose oxidase kits (Rsbio, Shanghai, China).

### 2.8. Glucose Uptake Assay

Glucose was measured using glucose uptake cell-based assay kit (Cayman Chemical Co., Ann Arbor, MI, USA). Briefly, C2C12 cells were seeded on a glass plate at 5 × 10^5^ cells/well and then differentiated with DMEM containing 2% horse serum for 5 days. Differentiated cells were then incubated with ARA extract (0.2, 0.5, or 1 mg/mL), metformin (2.5 mM), insulin (100 nM), or apigenin (50 *μ*M) in 1 mL of glucose-free medium containing 150 *μ*g/mL of 2-[N-(7-nitrobenz-2-oxa-1,3-diazol-4-yl) amino]-2-deoxy-d-glucose (NBDG) for 4 h, when supernatants were removed and 200 *μ*L of cell-based assay buffer was added per well. The amount of 2-NBDG taken up by cells was determined by fluorescence microscopy (Leica Biosystems, Wetzlar, Germany).

### 2.9. Free Fatty Acid Assay

C2C12 cells were differentiated in a 96-well plate for 5 days and then treated with ARA extract or metformin for 24 h. Free fatty acid (FFA) contents were estimated using a FFA quantification kit (Abcam, Eugene, OR, USA). In this assay, fatty acids are converted to their CoA derivatives and then oxidized to produce colored derivatives. FFA contents were determined colorimetrically using a spectrophotometer at *λ* = 570 nm.

### 2.10. Statistical Analysis

GraphPad Prism (GraphPad Software, Inc., San Diego, CA, USA) was used for the statistical analysis. Results are expressed as the means ± SEMs (standard errors of means) of three separate experiments and were analyzed using analysis of variance (ANOVA) followed by Tukey's test for multiple comparisons. Statistical significance was accepted for *p* values < 0.05.

## 3. Results

### 3.1. ARA Induced the Expression of PGC1*α*

**Figure 1:**
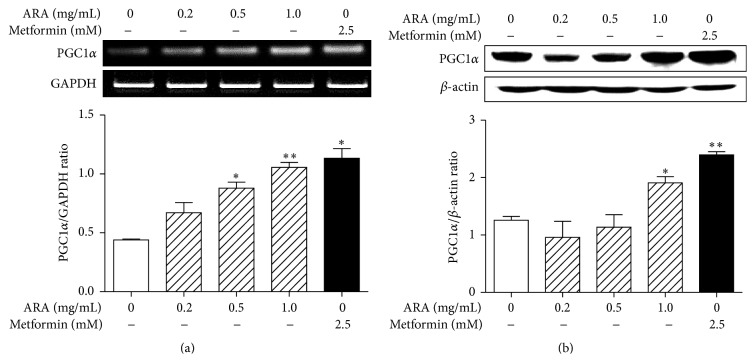
Effect of ARA extract on the expression of PGC1*α* in C2C12 cells. C2C12 cells were differentiated with DMEM containing 2% horse serum for 5 days and then treated with ARA extract (0.2, 0.5, and 1.0 mg/mL) or metformin (2.5 mM) for 24 h. PGC1*α* mRNA (a) and protein (b) levels were determined by RT-PCR and Western blotting, respectively. Values in histogram are the means ± SEMs of three independent experiments. ^*∗*^ *p* < 0.05 and ^*∗∗*^ *p* < 0.01 versus nontreated differentiated cells.

PGC1*α* is a key factor in mitochondrial biogenesis and plays an important role in fatty acid oxidation and thermogenesis in skeletal muscle [9]. To investigate the effect of ARA extract on the expression of PGC1*α* in C2C12 cells, we measured the mRNA and protein levels by RT-PCR and Western blotting, respectively. Treatment with ARA extract at 0.5 and 1.0 mg/mL significantly increased the levels of PGC1*α* mRNA (Figure 1(a)) and protein (Figure 1(b)) in C2C12 myotubes, as well as metformin at 2.5 mM.

### 3.2. ARA Regulated the Expressions of Mitochondrial Biogenesis/Thermogenesis-Related Factors and ATP Content

**Figure 2:**
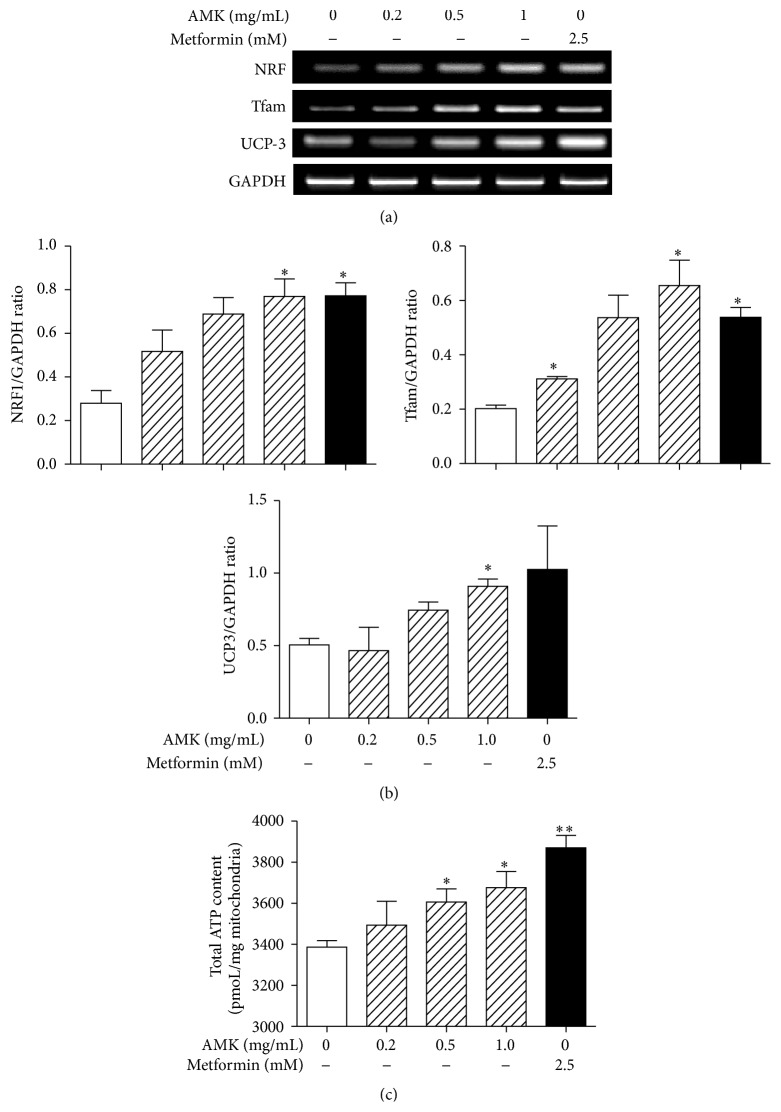
Effect of ARA extract on mitochondrial biogenesis/thermogenesis-related factors and ATP content. C2C12 cells were differentiated with DMEM containing 2% horse serum for 5 days and then treated with ARA extract (0.2, 0.5, and 1.0 mg/mL) or metformin (2.5 mM) for 24 h. The expressions of NRF, TFAM, and UCP3 were analyzed by RT-PCR (a). The values shown are ratios of mRNA GAPDH blot densities and are the means ± SEMs of three independent experiments (b). The total ATP content was measured using ATP calorimetric assay kit (c). Values in histogram are the means ± SEMs of three independent experiments. ^*∗*^ *p* < 0.05 and ^*∗∗*^ *p* < 0.01 versus nontreated differentiated cells.

NRF1 and TFAM are crucial factors of mitochondrial biogenesis in skeletal muscle, and PGC1*α* is their coactivator [8, 9]. Therefore, we examined the effects of ARA extract on the expression of NRF1 and TFAM gene in C2C12 cells by RT-PCR. ARA extract was found to increase the expressions of NRF1 and TFAM in a dose-dependent manner. Metformin also showed a similar effect (Figure 2(a)).

PGC1*α* also induces the expression of the mitochondrial protein, UCP3, which dissipates energy as heat and affects energy metabolism in skeletal muscle [24]. Therefore, we next investigated the effect of ARA extract on UCP3 expression in C2C12 cells. ARA extract at dose of 1.0 mg/mL significantly increased the expression of UCP3 mRNA in C2C12 myotubes (Figure 2(b)).

ATP is formed exclusively in mitochondria; ATP synthesis declines in concert with a reduction of key factors regulating mitochondrial biogenesis (PGC1*α*, NRF1, etc.) [25]. Therefore we analyzed the total ATP content in C2C12 myotubes after ARA treatment. ARA significantly increased ATP content (Figure 2(c)).

### 3.3. ARA Extract Activated the Phosphorylations of AMPK and ACC

**Figure 3:**
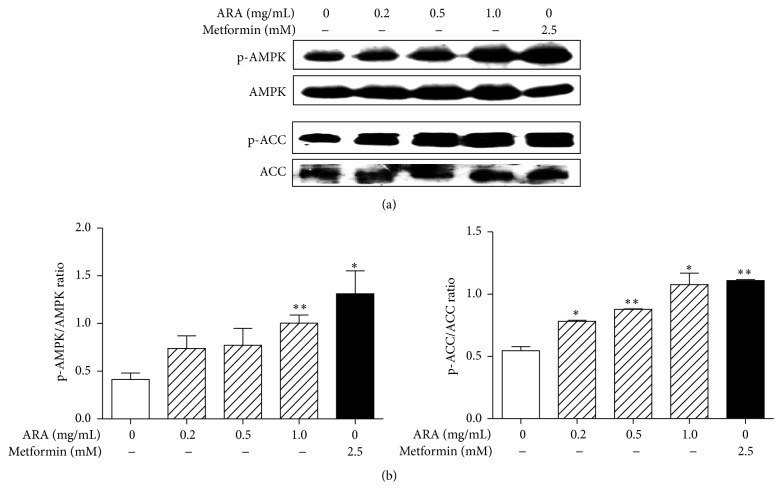
Effect of ARA extract on the phosphorylation of AMPK in C2C12 cells. C2C12 cells were differentiated with DMEM containing 2% horse serum for 5 days and then treated with ARA extract (0.2, 0.5, and 1.0 mg/mL) or metformin (2.5 mM) for 45 min. pAMPK, AMPK, pACC, and ACC protein levels were assessed by Western blotting (a) versus *β*-actin (the internal control). Values are expressed as the means ± SEMs of three independent experiments (b). ^*∗*^ *p* < 0.05 and ^*∗∗*^ *p* < 0.01 versus nontreated differentiated cells.

The phosphorylation of AMPK increases mitochondrial biogenesis, glucose transport, and fatty acid oxidation in skeletal muscle [8, 10]. Specifically, AMPK phosphorylates PGC1*α* directly; it initiates the gene regulatory functions of AMPK on mitochondrial biogenesis, glucose, and fatty acid metabolism in skeletal muscle and inactivates ACC which inhibits fatty acid *β*-oxidation [26]. As shown in Figure 3, the treatment of ARA extract in C2C12 myotubes increased the phosphorylation of AMPK and ACC in a dose-dependent manner. Metformin also significantly increased the phosphorylation of AMPK and ACC. This result similarly corresponded with those of PGC1*α*, NRF, TFAM, and UCP3.

### 3.4. ARA Extract Activated the Expression of SIRT1

**Figure 4:**
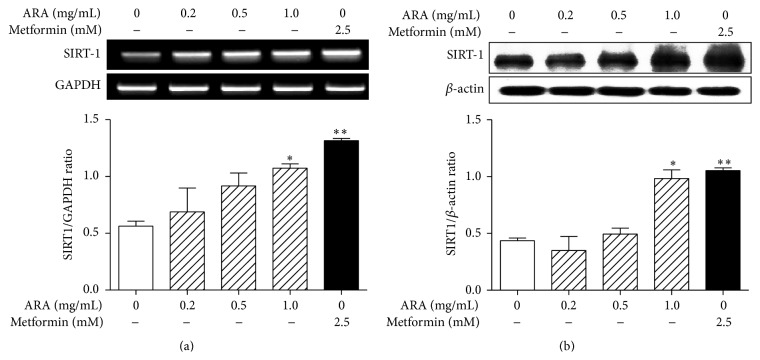
Effect of ARA extract on the expression of SIRT1 in C2C12 cells. C2C12 cells were differentiated with DMEM containing 2% horse serum for 5 days and then treated with ARA extract (0.2, 0.5, and 1.0 mg/mL) or metformin (2.5 mM) for 24 h. SIRT1 mRNA (a) and protein (b) levels were assessed by RT-PCR and Western blotting, respectively. Histogram values are the means ± SEMs of three independent experiments. ^*∗*^ *p* < 0.05 and ^*∗∗*^ *p* < 0.01 versus nontreated differentiated cells.

AMPK enhances the activity of SIRT1, another energy sensor, by increasing cellular NAD^+^ in skeletal muscle [27] and AMPK and SIRT1 act in concert with the master regulator of mitochondrial biogenesis, PGC1*α* [28]. Therefore, we examined the expression of SIRT1 mRNA and protein in C2C12 cells by RT-PCR and Western blotting, respectively. As shown in Figure 4, the expression of SIRT1 mRNA and protein was significantly increased by the treatment of ARA extract (1.0 mg/mL) and metformin (2.5 mM) in C2C12 cells.

### 3.5. ARA Increased Glucose Metabolism

**Figure 5:**
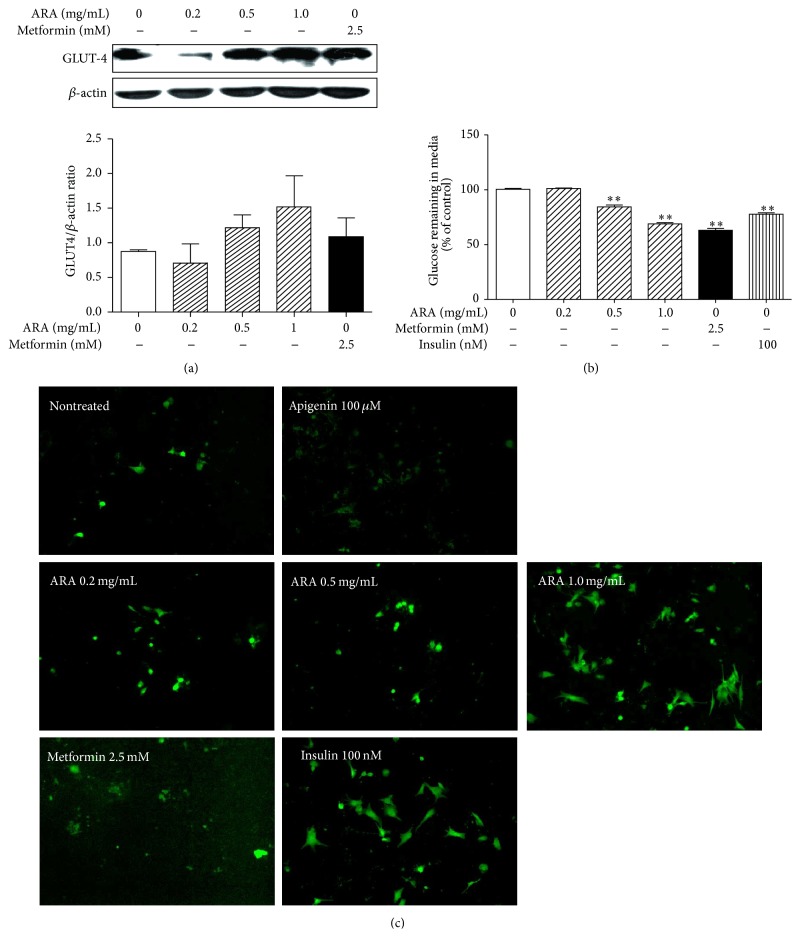
Effect of ARA extract on glucose metabolism in C2C12 cells. C2C12 cells were differentiated with DMEM containing 2% horse serum for 5 days and then treated with ARA extract (0.2, 0.5, or 1.0 mg/mL) or metformin (2.5 mM) for 24 h. GLUT-4 levels were assessed by Western blotting (a). Differentiated cells were treated with ARA extract, metformin, or insulin (100 nM) for 24 h and then glucose contents in media were determined using glucose oxidase kits (b). Differentiated cells were treated with ARA extract, metformin, insulin (100 nM), or apigenin (apigenin is known to decrease 2-NBDG uptake) (100 *μ*M) for 4 h. Glucose uptakes were observed by the amount of 2-NBDG taken up by cells in fluorescence microscopy (×100 original magnification) (c). Histogram values are the means ± SEMs of three independent experiments. ^*∗∗*^ *p* < 0.01 versus nontreated differentiated cells.

Glucose is a major oxidative substrate and the deregulation of glucose metabolism causes severe insulin resistance. In addition, mitochondrial function in skeletal muscle is essential for the maintenance of insulin sensitivity and glucose homeostasis [29]. Therefore, we investigated the expression of GLUT-4 as a glucose metabolism-related protein by Western blotting and conducted a glucose consumption assay and a glucose uptake assay. ARA extract at 0.5 and 1.0 mg/mL increased the expression of GLUT-4 protein in a dose-dependent manner (Figure 5(a)) and stimulated a glucose consumption (Figure 5(b)). Also, ARA extract dose-dependently elevated a glucose uptake and, in particular, at dose of 1.0 mg/mL, it was similar with that of insulin at 100 nM (Figure 5(c)).

### 3.6. ARA Extract Decreased FFA Levels and Increased Fatty Acid Oxidation

**Figure 6:**
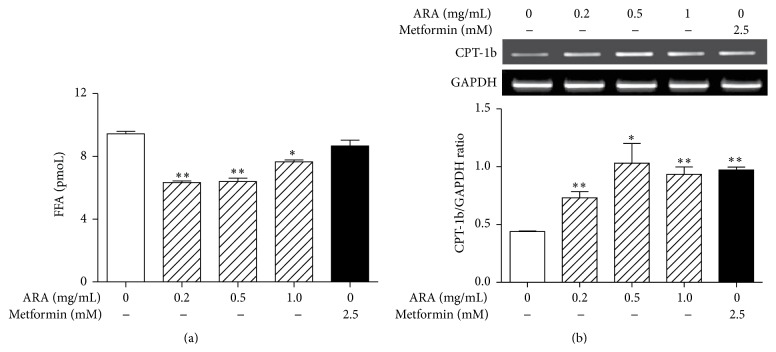
Effects of ARA extract on free fatty acid levels and CPT-1b expression in C2C12 cells. C2C12 cells were differentiated with DMEM containing 2% horse serum for 5 days and then treated with ARA extract (0.2, 0.5, and 1.0 mg/mL) or metformin (2.5 mM) for 24 h. FFA contents were measured in supernatants using a FFA assay (a). CPT-1b levels were assessed by RT-PCR (b). Histogram values are the means ± SEMs of three independent experiments. ^*∗*^ *p* < 0.05 and ^*∗∗*^ *p* < 0.01 versus nontreated differentiated cells.

Impaired mitochondrial function may contribute considerably to intramyocellular lipid accumulation, which has been linked to insulin resistance [30] and, thus, *β*-oxidation of FFAs in skeletal muscle has been identified as a treatment target [31]. Furthermore, CPT-1b is believed to be the rate-limiting step in this process [32]. Therefore, we investigated whether ARA extract decreased FFA and the expression of CPT-1b mRNA in C2C12 myotubes using FFA assay and Western blotting, respectively. As a result, ARA extract at 0.2, 0.5, and 1.0 mg/mL significantly inhibited FFA accumulation in C2C12 cells (Figure 6(a)) and also significantly increased the expression of CPT-1b gene (Figure 6(b)).

## 4. Discussion

Mitochondrial dysfunction appears to make a key contribution to insulin resistance, and thus, targeting the regulation of mitochondrial function could improve insulin resistance and associated metabolic diseases [33]. In this study, we focused on ARA extract as a natural medicine and its regulation of mitochondrial biogenesis in skeletal muscle, and, thus, we investigated the effects of ARA extract on the PGC1*α*-AMPK-SIRT1 pathway, which plays a central role in mitochondrial function and energy metabolism.

Metformin is an antidiabetic drug and AMPK activator and increases the expression of PGC1*α* via AMPK phosphorylation in skeletal muscle. In addition, it is known that metformin increases insulin sensitivity, fatty acid oxidation, and glucose uptake via this mechanism [34].

PGC1*α* and some of its targets, such as NRF-1 and TFAM, are main regulators of mitochondrial function and biogenesis in skeletal muscle [35]. In the present study, ARA extract increased the expression of PGC1*α* and downregulated its targets, NRF1 and TFAM, in C2C12 myotubes. In addition, ARA extract also induced the expression of UCP3, which is expressed predominantly in skeletal muscle. Furthermore, UCP3 has been involved in whole-body energy metabolism and in the regulations of ROS in the context of mitochondrial fatty acid transport and glucose metabolism in skeletal muscle [24]. To determine the effects of mitochondrial biogenesis associated with activation of these key factors, we measured the total ATP content. ARA also significantly increased ATP content. These results indicate that ARA extract can regulate mitochondrial function and biogenesis in skeletal muscle.

Metabolic sensors, such as AMPK and SIRT1, which act as a PGC1*α* gatekeeper, constitute vital links in the network that regulates metabolic homeostasis. In the present study, ARA extract increased the phosphorylation of AMPK and the expression of ACC as an AMPK substrate and induced the expression of SIRT1. These results suggest that ARA extract regulates mitochondrial function by activating of energy sensing molecules, including AMPK and SIRT1, in skeletal muscle. Moreover, the PGC1*α*-AMPK-SIRT1 pathway is connected with fatty acid oxidation and glucose uptake in mitochondria of skeletal muscle [36], and the oxidation of fatty acids and glucose in mitochondria largely accounts for ATP generation in skeletal muscles. In the present study, ARA extract increased glucose consumption, glucose uptake, and protein of GLUT4 in C2C12 myotubes. In addition, ARA increased the expression of CPT-1b and decreased FFA levels. These results provide that ARA might promote glucose metabolism and fatty acid oxidation in the mitochondria of skeletal muscle.

In previous studies, ARA extract was found to reduce body weights and serum triglyceride levels in HFD-induced obese rats [21] and to stimulate the insulin signaling pathway in 3T3-L1 adipocytes [22]. In the present study, ARA was found to modulate lipid and glucose metabolism via the metabolic energy sensors PGC1*α*, AMPK, and SIRT1 in C2C12 myotubes. These findings suggest that the mechanism underlying the antiobesity effect of ARA extract is due to enhanced insulin sensitivity. Atractylenolide III is a major biological active component in ARA extract [37] and was analyzed by HPLC (Supplementary Figure  1). In the future, we will investigate the effects of compounds on C2C12 myotubes to determine whether they are responsible for the regulatory effect of ARA on energy metabolism and mitochondrial function.

## 5. Conclusions

ARA extract was found to stimulate mitochondria biogenesis markers including PGC1*α*, NRF1, and TFAM with increase of ATP content. It also activated AMPK and SIRT1, the energy sensing molecules, while, in turn, ARA extract regulated glucose and fatty acid metabolism in C2C12 myotubes. Our findings suggest that ARA extract and its active constituents have therapeutic potential for the treatment of insulin resistance, obesity, and T2 diabetes.

## Supplementary Material

HPLC Pattern of ARA Extract. ARA extract was determined directly using an HPLC system equipped with an Aminex-87H column and UV detector (A). Atractylenolide III was used as a standard compound (B).
