# Supplementary figures and images for: The Roots of Atractylodes macrocephala Koidzumi Enhanced Glucose and Lipid Metabolism in C2C12 Myotubes via Mitochondrial Regulation
Source: Evid Based Complement Alternat Med. 2015 Nov 4;2015:643654. doi: 10.1155/2015/643654 (PMC4649076; doi:10.1155/2015/643654)

Supplementary figure 1. HPLC fingerprintings of ARA extract and Atractylenolide III

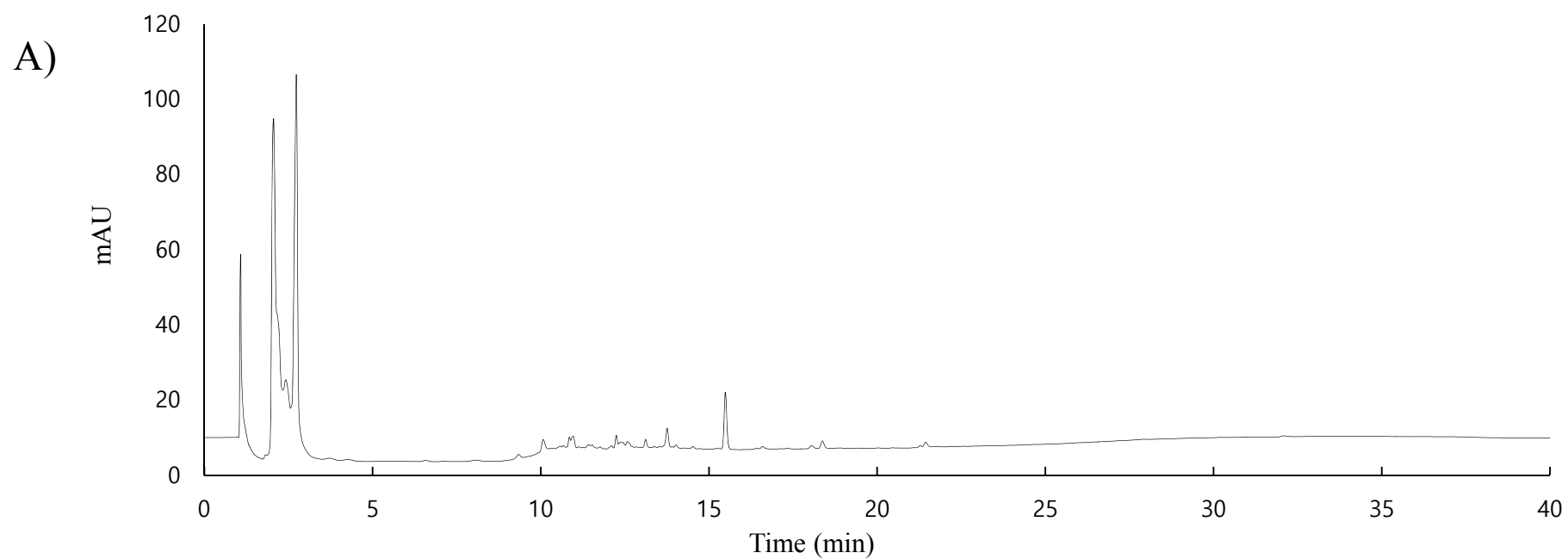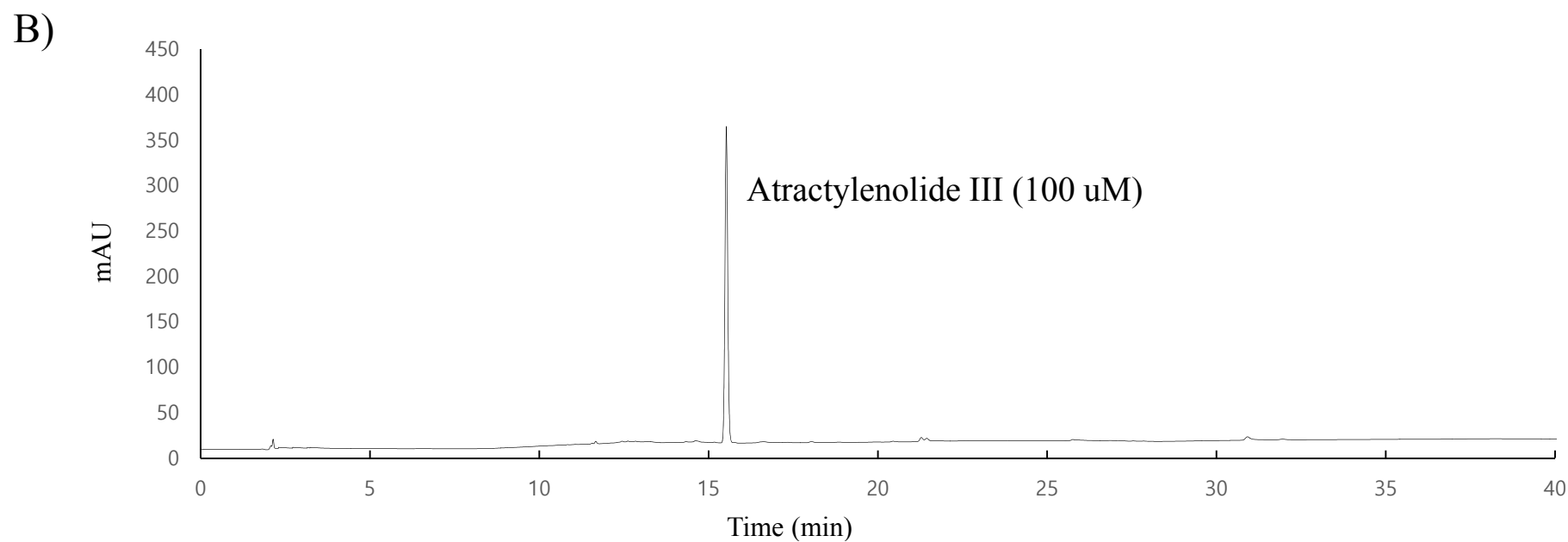

Supplement: Supplementary file 1 — HPLC Pattern of ARA Extract. ARA extract was determined directly using an HPLC system equipped with an Aminex-87H column and UV detector (A). Atractylenolide III was used as a standard compound (B). [file 643654.f1.pdf]
